# Expression of senescence-related genes in human corneal endothelial cells

**Published:** 2008-01-29

**Authors:** Zhenhua Song, Ye Wang, Lixin Xie, Xinjie Zang, Hongmei Yin

**Affiliations:** State Key Lab Cultivation Base, Shandong Provincial Key Lab of Ophthalmology, Shandong Eye Institute, Qingdao, China.

## Abstract

**Purpose:**

To investigate the expression of *p16^INK4a^*, *p21^WAF1/CIP1^*, *p27^KIP1^*, and *p53* in human corneal endothelial cell (HCEC) senescence ex vivo at various donor ages.

**Methods:**

Residual corneal tissues obtained after penetrating keratoplasty were used in this study. Age, death-to-preservation interval, and preservation-to-surgery interval of the donors were recorded. Corneal endothelial cell survival and density were evaluated by trypan blue and alizarin red staining immediately after keratoplasty. Fresh frozen sections of donor corneas at various ages (18, 33, 54, and 68 years) were immunostained in situ. Total RNA extracted from age groups of 20, 30, 40, 50, and 60 years was evaluated by reverse-transcriptase polymerase chain reaction (PCR) to reveal the expression of the senescence-related genes, *p16^INK4a^*, *p21^WAF1/CIP1^*, *p27^KIP1^*, and *p53*, in HCECs. Total RNA extracted from 20-, 24-, 26-, 30-, 50-, 55-, 56-, and 60-year-old donor groups was subjected to real-time PCR analysis for measurement of gene expression. The results of the young (≤30 years) and the old (≥ 50 years) were compared by the unpaired *t*-test. Ex vivo senescence of HCECs from the donors at various ages (9, 17, 23, 57, 65, and 67 years) was observed by senescence-associated β-galactosidase activity (SA-β-Gal) staining at pH 6.0.

**Results:**

The mean endothelial cell density of the donor corneas was 2,391.4±84.6 cells/mm^2^, and the survival rate of the endothelial cells was 84.4%±5.3%. Hematoxylin and eosin staining showed normal structures of the corneal epithelium, stroma, and endothelium. The expression and nuclear localization of p16^INK4a^, p21^WAF1/CIP1^, p27^KIP1^, and p53 in HCECs were confirmed by immunohistochemistry in situ. Reverse transcriptase PCR examination showed positive target bands of each gene at each age group. An age-related increase in *p16^INK4a^* expression was observed by real-time PCR (p=0.014). There was no significant difference in the expression levels of *p21^WAF1/CIP1^*, *p27^KIP1^*, and *p53* between the young and old donors (p=0.875, 0.472, and 0.430, respectively). Strong SA-β-Gal activity was observed in the endothelial cells of the old donors while there was weak and little-to-no blue staining in the endothelia from the young.

**Conclusions:**

The population of HCECs exhibiting senescence-like characteristics increases with age. *p16^INK4a^*, *p21^WAF1/CIP1^*, *p27^KIP1^*, and *p53* are expressed in HCECs despite donor ages. The p16^INK4a^ signal pathway might play a key role in the process of senescence in HCECs.

## Introduction

The corneal endothelium is a single layer of cells located at the posterior of the cornea. It is responsible for maintaining corneal transparency through its barrier and ionic pump function. Human corneal endothelial cells (HCECs) are considered to be nonproliferative in vivo, and therefore, a corneal endothelial wound heals predominantly by cell migration and enlargement [1-3]. It is clear that HCECs do retain proliferative capacity in vivo [4-6]. However, up to now, methods to induce proliferation in cultured HCECs, such as viral oncogene transformation [7] and growth factor supplement [8], cannot be used in clinical practice. As a result of excessive cell loss due to accidental or surgical trauma or disease, corneal endothelial dysfunction may lead to corneal edema and vision loss, requiring corneal transplantation to restore normal vision. However, graft endothelial cell density (ECD) even without a rejection episode continues to decrease at a rapid rate compared to normal eyes [9,10]. Correspondingly, late endothelial failure [9,10] and chronic corneal allograft dysfunction (CCAD) [11] may occur, which has become a major cause of late transplant failure [10,12]. Some studies have reported that the deterioration in organ function might be related with the senescence of key cells within that tissue, and the chronic transplant dysfunction was considered to be caused by accelerated aging [13-15]. Is it accelerated senescence of HCECs resulting from trauma or disease that contributes to the dysfunction of corneal endothelial cells? An investigation on the senescence of normal HCECs is required first.

Cellular senescence is a program activated by normal cells in response to a variety of stresses. Many studies have reported the importance of senescence-related genes such as *p16^INK4a^*, *p21^WAF1/CIP1^*, *p27^KIP1^*, and *p53* in cellular senescence. *p16^INK4a^*, *p21^WAF1/CIP1^*, and *p27^KIP1^* are cyclin-dependent kinase inhibitors (CDKI), which are negative regulators of the cell cycle. They can inhibit the cell cycle progression and maintain the G_1_ phase arrest of cells [16]. Their accumulation in aged cells of some types was also reported [17,18]. p53 functions as a central integration point for various signaling pathways of senescence and inhibits cell division primarily through a p21^WAF1/CIP1^ pathway. Inactivation of p53 may prevent or delay the onset of senescence. p53 could not only downregulate cell division during the normal process of aging but also get involved in a response to telomere shortening [19,20], oxidative stress, and DNA damage [21,22]. A different signaling pathway may be activated in different species or by various types of stresses. It was reported that p16^INK4a^, p21^WAF1/CIP1^, p27^KIP1^, and p53 are all expressed in corneal endothelial cells of several species [23-25]. Their different age-related expressions in cultured HCECs might contribute to the age-related difference in the proliferative capacity [5,26]. However, development of aging in HCECs in vivo has not yet been proven because of the influence of the culture environment and passage stress. Obviously, the role of HCEC senescence in different clinical problems cannot be elucidated without an evaluation on the mechanisms of HCEC aging in vivo. To investigate the key effectors in the mechanism of HCEC senescence in vivo, the relative expression of senescence-related genes was analyzed in HCEC ex vivo at various donor ages in this study.

## Methods

### Human corneal tissues and evaluation

Corneal rims, residual parts dissected from donor corneas by a circular trephine in penetrating keratoplasty, were immediately collected for this study. One quarter of each corneal rim was cut and stained with trypan blue and alizarin red [27]. Donor corneal rims with an ECD of < 2000 cells/mm^2^ or an endothelial cell survival rate of < 80% were excluded [28]. Additional exclusion criteria were similar to those previously indicated [29]. Some corneal rim tissues were placed with the endothelium side up on a Teflon block and cut with a circular trephine of 10–11 mm in diameter within the Schwalbe’s line under a surgical microscope. Descemet’s membrane with endothelium was easily detached from the underlying stroma with forceps and stripped away intact. The stripped endothelial tissues were frozen at −80 °C for RNA analysis. Some corneal rims were sectioned and frozen in optimum cutting temperature compound (Tissue-Tek, Tokyo, Japan) and stored at −80 °C until ready for cryostat sectioning.

Before surgery, the human donor corneas were provided by the Shandong Eye Institute Eye Bank (Qingdao, China) and preserved in DX intermediate-term medium at 4 °C [30]. Most of the donors suffered from sudden death. The death-to-preservation interval (< 20 h) and preservation-to-surgery interval (< 4 days) were recorded. The handling of the donor tissues adhered to the tenets of the Declaration of Helsinki of 1975 and its 1983 revision in protecting donor confidentiality.

**Table 1:** Primers used for reverse-transcriptase polymerase chain reaction

Gene name	Primer sequence	Product length
p16^INK4a^	F: GAC ATC CCC GAT TGA AAG AA	198 bp
	R: TTT ACG GTA GTG GGG GAA GG	
p21^WAF1/CIP1^	F: GAC ACC ACT GGA GGG TGA CT	172 bp
	R: CAG GTC CAC ATG GTC TTC CT	
p27^KIP1^	F: TCT ACT GCG TGG CTT GTC AG	240 bp
	R: CTG TAT TTG GAG GCA CAG CA	
p53	F: GGC CCA CTT CAC CGT ACT AA	156 bp
	R: GTG GTT TCA AGG CCA GAT GT	
GAPDH	F: ACC ACA GTC CAT GCC ATC AC	439 bp
	R: TCC ACC ACC CTG TTG CTG TA	

Primers are used to detect genes from mRNA in human corneal endothelial cells at various ages. The sequence for design of forward and reverse primers and the number of base pairs in expected polymerase chain reaction product are shown.

### Qualitative immunohistochemical detection

Streptavidin-biotin complex (SABC) immunohistochemical stainings of p16^INK4a^, p21^WAF1/CIP1^, p27^KIP1^, and p53 were qualitatively detected in sections of four corneal samples (18, 33, 54, and 68 years). Transverse corneal sections (6 μm) were put on slides and air dried. Slides were fixed in acetone at −20 °C for 10 min and washed in PBS (pH 7.4). Endogenous peroxidase activity was reduced in the section by an incubation in methanol with 0.6% hydrogen peroxide for 30 min. Sections were incubated with 5% BSA supplied in an instant-type SABC immunohistochemistry kit (Boster Corp, Wuhan, China) for 20 min then with rabbit polyclonal anti-p16, mouse monoclonal anti-p21, mouse monoclonal anti-p27, or rabbit monoclonal anti-p53 antibodies (1:50, 1:100, 1:50, and 1:25, respectively; Lab Vision Corp, Fremont, CA) diluted in antibody dilution buffer (Boster Corp) overnight at 4 °C. After the PBS washing, biotin-conjugated anti-mouse or anti-rabbit immunoglobulin G, depending on the primary antibody, was used as a secondary antibody at 37 °C for 20 min. The sections were then washed with PBS and treated with SABC solution at 37 °C for 20 min. Peroxidase activity was detected with the substrate diaminobenzidine (DAB kit; Boster Corp). Negative controls for all antigens consisted of tissue sections incubated with PBS instead of a primary antibody. The slides were treated with graded alcohols and xylene and mounted with DPX (distyrene, plasticizer, and xylene; BDH, Poole, England). Permanent slides were covered with a 1.5-mm thick cover slip, examined using a light microscope (Eclipse e800; Nikon, Tokyo, Japan), and photographed with a Nikon digital camera DXM 1200.

### Qualitative reverse transcriptase-polymerase chain reaction examination

Samples of HCEC tissue from donors of various ages were divided into groups of 20, 30, 40, 50, and 60 years. Three to four corneal rims of similar age were included in each group. Total RNA was isolated from HCECs of each group using the RNeasy Mini kit (Qiagen GmbH, Hilden, Germany) according to the manufacturer’s protocol. The polymerase chain reaction (PCR) primers were designed on the basis of the published human gene sequences (Table 1). Reverse-transcriptase PCR (RT–PCR) was performed to evaluate the expression of these genes in HCECs using the housekeeping gene, *GAPDH*, as an internal control. In brief, the first-strand cDNA was synthesized from equal amounts of total RNA (300–500 ng/μl) using a cDNA synthesis kit (BBI, Toronto, Canada) according to the manufacturer’s protocol. PCR amplification was performed (Mastercycler gradient; Eppendorf, Köln, Germany) using the following program: denaturation at 95 °C for 2 min followed by 35 cycles of denaturation at 95 °C for 1 min, annealing at 60 °C for 30 s, and extension at 72 °C for 1 min followed by a last extension at 72 °C for 10 min. The negative controls consisted of omission of RNA or reverse transcriptase from the reaction mixture. An aliquot of the PCR product was electrophoresed on 1.5% agarose gels and stained with ethidium bromide for 40 min. Photographs of the gel were taken with a high-resolution camera. The density of the bands was analyzed on the gel using ultraviolet (UV) illumination and Image J analysis software.

### Fluorescent real-time quantitative polymerase chain reaction

Samples were divided into age groups of 20, 24, 26, 30, 50, 55, 56, and 60 years. Three to four corneal rims of similar age (within two years) were included in each group. For the pooled donors, the average age was determined. The same procedures of total RNA extraction and first-strand cDNA synthesis were performed as mentioned above. Taqman reagents and the ABI Prism 7500 System were used in real-time PCR as recommended by the manufacturer (Applied Biosystems, Foster City, CA). Each sample was assayed in duplicate with Taqman Universal PCR Master Mix (Applied Biosystems). Primers and oligonucleotide probes used are listed in Table 2. The cycling conditions were as follows: 10 min at 95 °C and 40 cycles of amplification for 15 s at 95 °C and 1 min at 60 °C. The expected fragment length was between 150 and 300 bp. The quantification data were analyzed with the SDS System Software (7500 System; Applied Biosystems). After PCR baseline subtraction was performed by the software, the log-linear portion of the fluorescence versus cycle plot was extended to determine a fractional cycle number at which a threshold fluorescence was obtained (threshold cycle, C_t_) as a reference for each analyzed gene and *GAPDH*. The comparative C_t_ method was used for quantification of the target genes relative to *GAPDH*. The 20-year-old group was used as a calibrator, and the data were presented as the fold change in gene expression relative to the calibrator. The difference in the means between the young (≤ 30 years) and old groups (≥ 50 years) was calculated with an unpaired *t*-test (SPSS software version 12.0; SPSS Inc., Chicago, IL). A p<0.05 was considered to be significant.

**Table 2:** Primers and probes used for fluorescent real-time quantitative polymerase chain reaction

Gene name	Primer sequence(5′→3′)
p16^INK4a^	F: AAG GTC CCT CAG ACA TCC C
	R: TGT AGG ACC TTC GGT GAC TG
	PROBE: FAM-TCC GGA GGT TTC TCA GAG CCT CTC-TAMRA
p21^WAF1/CIP1^	F: CAG ACC AGC ATG ACA GAT TTC
	R: TTA GGG CTT CCT CTT GGA GA
	PROBE: ACC ACT CCA AAC GCC GGC TG
p27^KIP1^	F: CCT CCT CCA AGA CAA ACA GC
	R: CAT TCA GAG CGG GAT TAT CTT T
	PROBE: FAM-TCG AGT TCC TGA CAA GCC ACG C-TAMRA
p53	F: AAC AAC ACC AGC TCC TCT CC
	R: CTC ATT CAG CTC TCG GAA CA
	PROBE: FAM-CCC TTC AGA TCC GTG GGC GT-TAMRA
GAPDH	F: ATG CTG GCG CTG AGT ACG T
	R: AGC CCC AGC CTT CTC CAT
	PROBE: FAM-TGG AGT CCA CTG GCG TCT TCA-TAMRA

Fluorescent real-time quantitative polymerase chain reaction is performed as specified in the Methods section. Sequence specific primers and probes for *p16^INK4a^, p21^WAF1/CIP1^, p27^KIP1^, *and *p53* are listed.

### Senescence-associated β-galactosidase activity staining

According to previous reports [29,31], corneal rims obtained from six donors (9, 17, 23, 57, 65, and 67 years) were washed with PBS and fixed with 4% formaldehyde at room temperature for 15 min. After the wash with PBS (pH 6.0), the tissues were incubated at 37 °C overnight in a humidified chamber with freshly prepared SA-β-Gal staining solution (1 mg/ml X-Gal in dimethylformamide, 40 mmol/l citric acid and phosphate buffer, pH 6.0, 5 mmol/l potassium ferrocyanide, 5 mmol/l potassium ferricyanide, 150 mmol/l sodium chloride, and 2 mmol/l magnesium chloride). The positive control for this assay consisted of incubating corneal rims in staining solution adjusted to pH 4.0 as required for lysosomal β-galactosidase staining. On the following day, tissues were washed twice in PBS at room temperature for 10 min, and staining was visualized and captured using a microscope equipped with a digital camera (Eclipse e800; Nikon).

## Results

### Qualitative examination of senescence-related genes in human corneal endothelial cells

**Figure 1:**
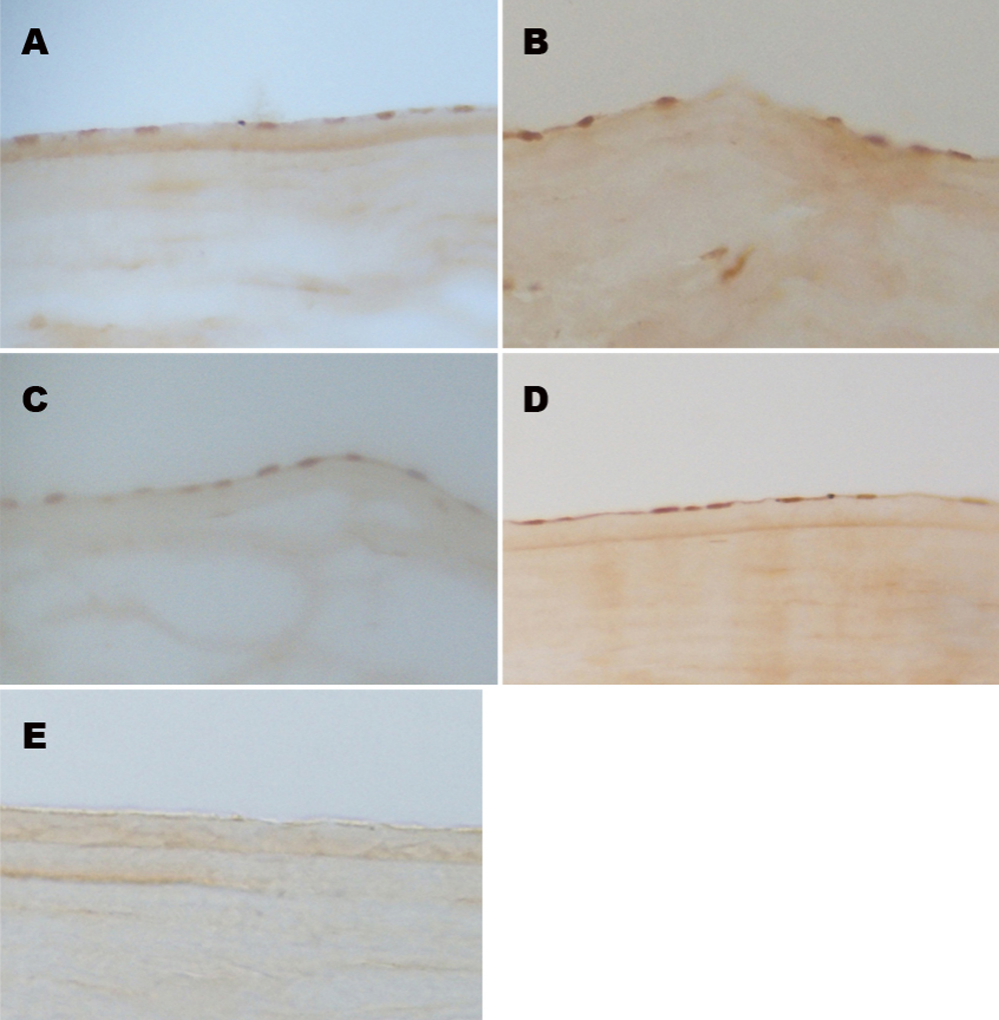
Immunohistochemical staining of *p16^INK4a^, p21^WAF1/CIP1^, p27^KIP1^,* and *p53. *Representative fresh-frozen sections of human corneas from donors at various ages (**A**: p16, 18 years; **B**: p21, 33 years; **C**: p27, 54 years; **D**: p53, 18 years; **E**: control, 68 years) are shown. Positive staining for all four senescence-related genes are clearly visible in the endothelial nuclei. Magnification; 400×.

**Figure 2:**
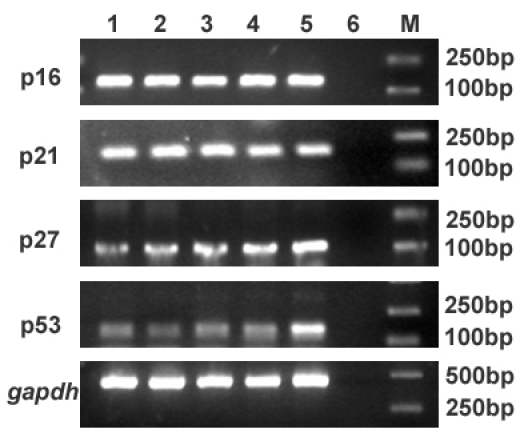
Detection of *p16^INK4a^*, *p21^WAF1/CIP1^*, *p27^KIP1^*, and *p53* mRNA. Electrophoretic gels show the presence of mRNA for the four senescence-related genes. Lane 1 to Lane 5 shows HCECs of the 20-, 30-, 40-, 50-, and 60-year-old groups, respectively. Lane 6 is the negative control. Lane M is Marker DL 2000. All bands migrate to their expected product length as summarized in Table 1.

Positive staining for p16^INK4a^, p21^WAF1/CIP1^, p27^KIP1^, and p53 were found and showed a similar nuclear localization in HCECs. Figure 1 presents representative micrographs of the immunohistochemical staining in situ of freshly frozen corneal sections. The analysis of mRNA from HCECs by RT–PCR indicated that *p16^INK4a^*, *p21^WAF1/CIP1^*, *p27^KIP1^*, and *p53* transcripts were present at various donor ages. No band was seen in the controls without RNA or reverse transcriptase (Figure 2).

### Quantitative analysis of relative expression of senescence-related genes in human corneal endothelial cells

**Figure 3:**
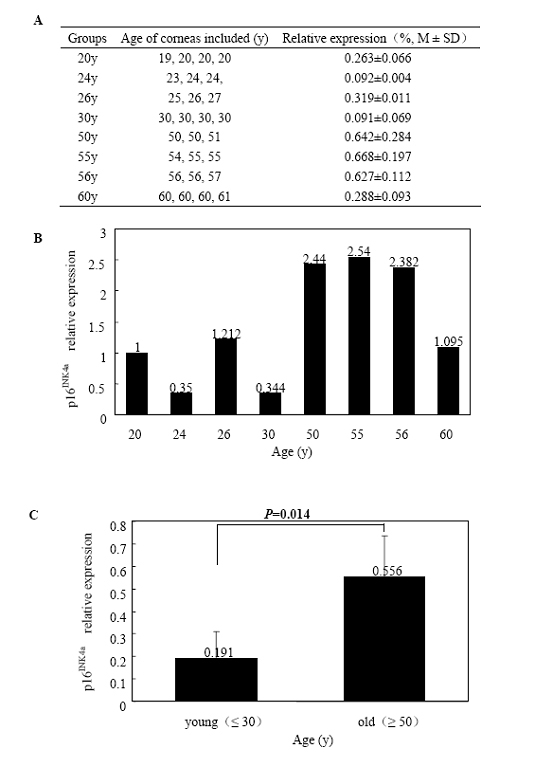
Quantitative real-time polymerase chain reaction analyses of *p16^INK4a^* mRNA expression in human corneal endothelial cells at various ages. Total RNA was isolated from HCECs of each group, and the first-strand cDNA were aynthesized from equal amounts of total RNA. Quantitative real-time PCR was performed to analyze *p16^INK4a^*mRNA expression. **A** shows the expression of *p16^INK4a^* from the eight age groups normalized to *GAPDH *mRNA. **B** demonstrates fold change in *p16^INK4a^* expression relative to the calibrator of the 20-year-old group. **C** is the comparison of the average level of *p16^INK4a^*mRNA expressed in HCECs between the young (≤ 30 years) and old (≥ 50 years) donors. Data are presented as mean±standard deviation, and a p<0.05 is considered to be statistically significant. Results indicate a significant increase (p=0.014) in *p16^INK4a^* mRNA expression in HCECs from the old donors.

**Figure 4:**
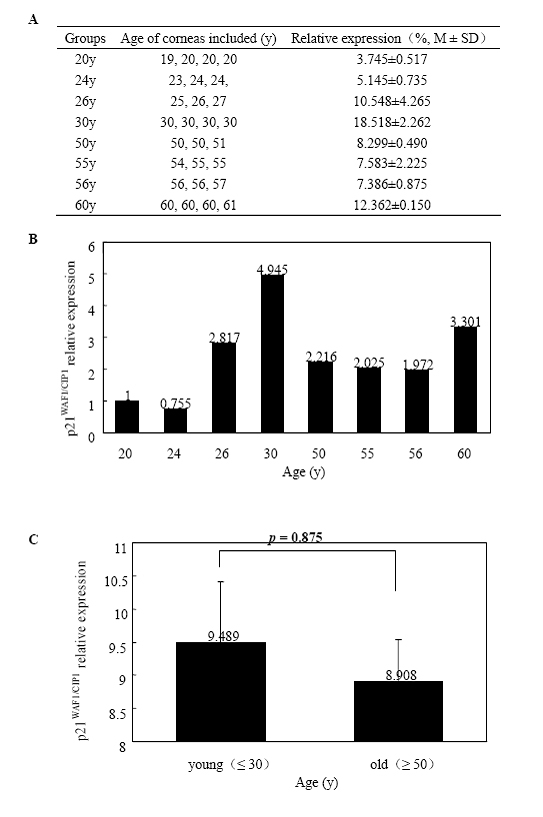
Quantitative real-time polymerase chain reaction analyses of *p21^WAF1/CIP1 ^*mRNA expression in human corneal endothelial cells at various ages. Total RNA was isolated from HCECs of each group, and the first-strand cDNA were aynthesized from equal amounts of total RNA. Quantitative real-time PCR was performed to analyze* p21^WAF1/CIP1^* mRNA expression. **A** shows the expression of *p21^WAF1/CIP1^* from the eight age groups normalized to *GAPDH *mRNA. **B** demonstrates fold change in *p21^WAF1/CIP1^* expression relative to the calibrator of the 20-year-old group. **C **is the comparison of the average level of *p21^WAF1/CIP1^* mRNA expressed in HCECs between the young (≤ 30 years) and old (≥ 50 years) donors. Data are presented as mean±standard deviation and a p<0.05 is considered to be statistically significant. Results indicate no significant increase (p=0.875) in *p21^WAF1/CIP1^* mRNA expression in HCECs from the old donors.

**Figure 5:**
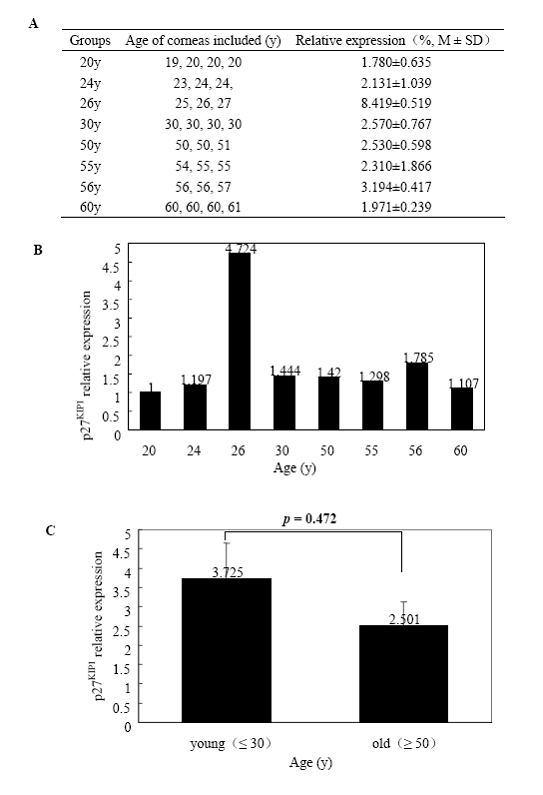
Quantitative real-time polymerase chain reaction analyses of *p27^KIP1^* mRNA expression in human corneal endothelial cells at various ages. Total RNA was isolated from HCECs of each group, and the first-strand cDNA were aynthesized from equal amounts of total RNA. Quantitative real-time PCR was performed to analyze* p27^KIP1^* mRNA expression. **A** shows the expression of *p27^KIP1^* from the eight age groups normalized to *GAPDH *mRNA. **B** demonstrates fold change in *p27^KIP1^* expression relative to the calibrator of the 20-year-old group. **C **is the comparison of the average level of *p27^KIP1^* mRNA expressed in HCECs between the young (≤ 30 years) and old (≥ 50 years) donors. Data are presented as mean±standard deviation, and a p<0.05 is considered to be statistically significant. Results indicate no significant increase (p=0.472) in *p27^KIP1^* mRNA expression in HCECs from the old donors.

**Figure 6:**
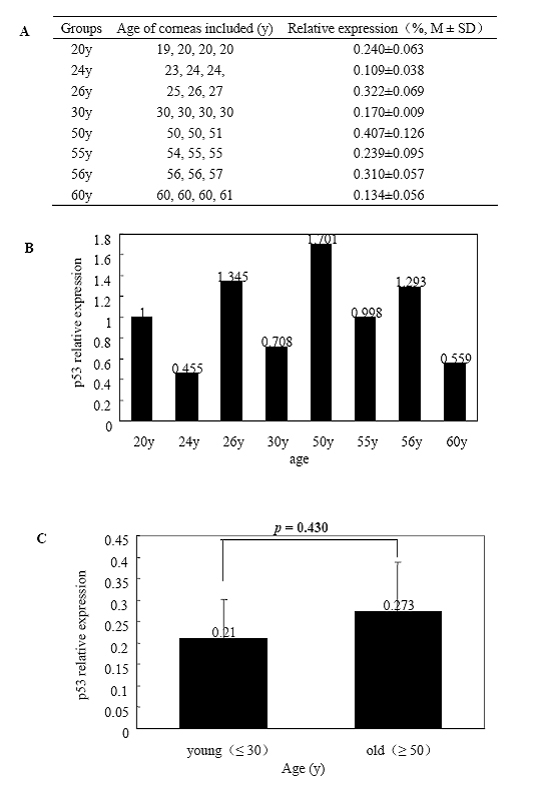
Quantitative real-time polymerase chain reaction analyses of *p53* mRNA expression in human corneal endothelial cells at various ages. Total RNA was isolated from HCECs of each group, and the first-strand cDNA were aynthesized from equal amounts of total RNA. Quantitative real-time PCR was performed to analyze* p53* mRNA expression. **A** shows the expression of *p53* from the eight age groups normalized to *GAPDH *mRNA. **B** demonstrates fold change in *p53* expression relative to the calibrator of the 20-year-old group. **C** is the comparison of the average level of *p53* mRNA expressed in HCECs between the young (≤ 30 years) and old (≥ 50 years). Data are presented as mean±standard deviation, and a p<0.05 is considered to be statistically significant. Results indicate no significant increase (p=0.430) in* p53* mRNA expression in HCECs from the old donors.

Total RNA extracted from HCECs of the eight age groups was subjected to quantitative real-time PCR for *p16^INK4a^*, *p21^WAF1/CIP1^*, *p27^KIP1^*, and *p53*. Figure 3 shows the expression of *p16^INK4a^* normalized to *GAPDH* RNA and the fold change in gene expression related to the calibrator of the 20-year-old group. There was a statistically significant difference in *p16^INK4a^* expression between the young (≤ 30 years) and old groups (≥ 50 years; p=0.014). Relative gene expression quantitation and fold change from the eight groups with *p21^WAF1/CIP1^*, *p27^KIP1^*, and *p53* were shown in Figure 4, 5, and 6, respectively. There was no significant difference in *p21^WAF1/CIP1^*, *p27^KIP1^*, and *p53* expression between any two age groups (p=0.875, 0.472, 0.430, respectively). HCECs from the old donors expressed significantly higher levels of *p16^INK4a^* than cells from young donors. In contrast, there were no obvious changes in the expression of *p21^WAF1/CIP1^*, *p27^KIP1^*, and *p53* with age.

### Evaluation of senescence-associated β-galactosidase activity in human corneal endothelial cells

**Figure 7:**
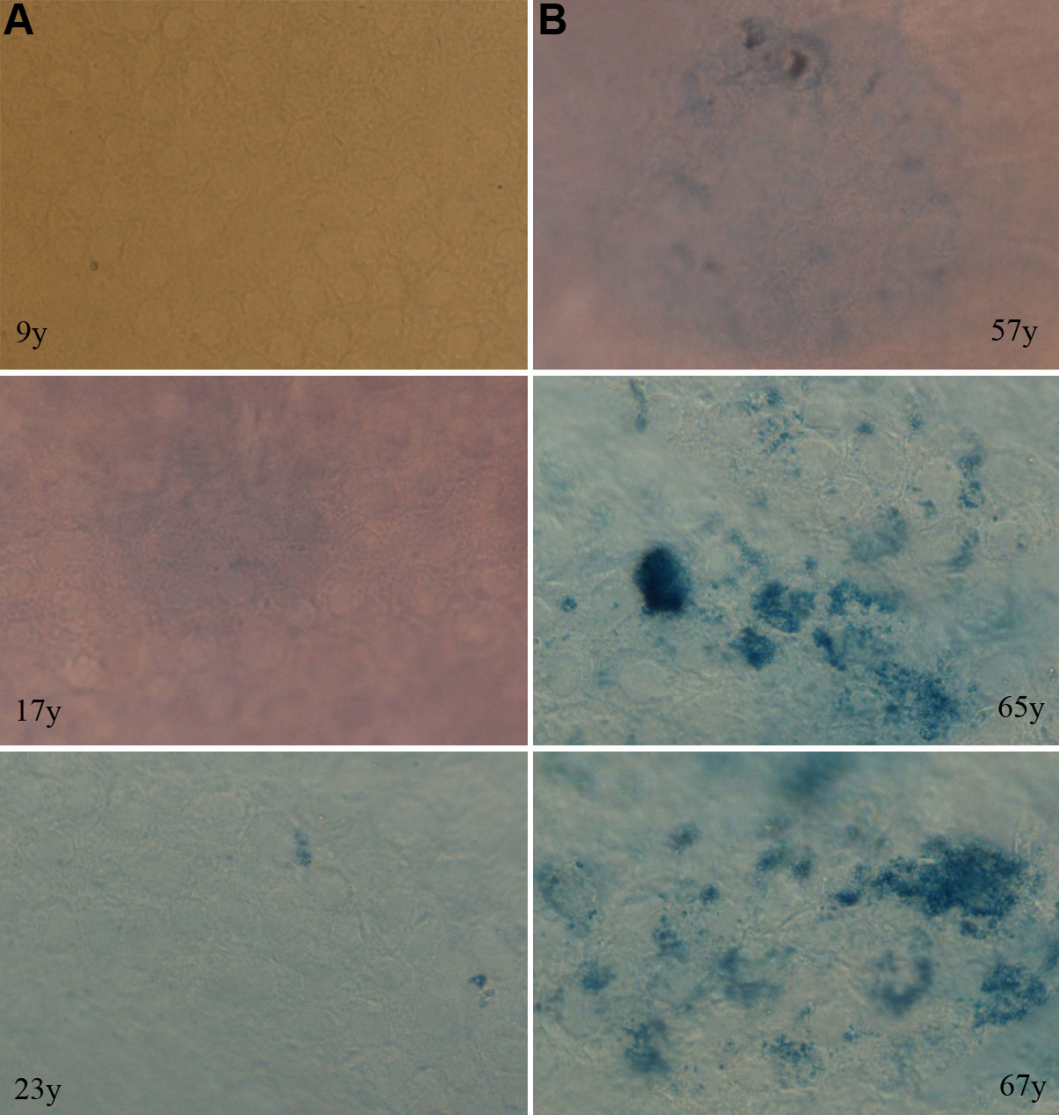
Senescence-associated β-galactosidase activity stainings in human corneal endothelial cells of ex vivo corneas from the young and old donors. In column **A,** there are weak and few to no stainings for SA-β-Gal activity in the young donor. In column **B,** more focal weak and multifocal intense stainings for SA-β-Gal are detected in the old donors. Magnification; 200×.

SA-β-Gal has been recommended as an indicator of cell senescence in vitro [32] and in vivo [33]. We assessed SA-β-Gal staining for HCECs of different ages. As shown in Figure 7A, there was weak blue staining for SA-β-Gal activity in HCECs from the young donors. On the contrary, a much more focal and even multifocal intense staining for SA-β-Gal activity was detected in HCECs from the old donors (Figure 7B). There was an increase in the number of HCECs that exhibited characteristics of senescence from the old donors. The positive staining was confirmed by normal β-galactosidase staining at pH 4.0 in all cells.

## Discussion

Penetrating keratoplasty remains a major choice in the treatment of corneal endothelial dysfunction. However, besides the lack of donors, poor quality of the donor corneas with low cell density brings limits to the surgical involvement. Moreover, corneal grafts may lose endothelial cells at an accelerated rate compared with healthy corneas [9,10]. This loss eventually leads to late endothelial failure of the corneal graft, which is the major overall reason for graft failure [10,12]. ECD is a common core factor related to corneal endothelial cell diseases, donor cornea quality, and graft prognosis. The deterioration of organ or graft function was considered to be the result of accelerated senescence of key cells within that tissue [13-15]. Therefore, we can suppose the importance of senescence of corneal endothelium on ECD.

Among the morphological characteristics of senescent cells are cell enlargement, increased vacuolation, and the presence of lipofuscin. Cells also exhibit proliferative changes, including presence of multiple nuclei and/or greater than 2N DNA, a decreased response to mitogens, decreased telomere length, an increase in expression of the tumor suppressor, *p53*, and suppression of *CDKI* genes. These morphological and proliferative characteristics of senescent cells can be observed in HCECs at aging [34-36] or damage [36], after transplantation [37], or when cultured in vitro [15], which may support the likelihood of HCEC senescence. Although HCECs have long telomeres throughout life [38], the process of cell senescence is very complex and most likely determined by three interdependent or convergent mechanisms [39,40]: the intracellular biological clock—telomeres, genetic mechanisms of regulation, and the influence of various environmental stress factors.

Neutral (pH 6.0) β-galactosidase, termed SA-β-Gal, is widely used in the identification of the presence of senescent cells in vitro [32] and in vivo [33]. Its frequency has been shown to rise with the increasing age of several tissues from humans and rodents. Mishima et al. [31] first applied SA-β-Gal staining to eye research. Recently, it was used in detecting the characteristics of HCEC senescence by Mimura and Joyce [29]. In this confirmative study, there was little to no staining for SA-β-Gal in HCECs from the young donors but relatively more intense and greater staining from the old. It proves that senescent HCECs accumulate SA-β-Gal with age in vivo and confirms what Mimura and Joyce found [29].

There were few reports on the importance of *CDKI* genes in senescence of HCECs in vitro. The role of cell senescence in clinical eye problems, such as late endothelial failure, cannot be elucidated without an evaluation on the mechanism of HCECs aging in vivo. Primary culture cells are considered to possess similar biological characters with those in vivo. However, the expression of *CDKI* in HCECs may be influenced by cell replication in the culture microenvironment in vitro. The only evaluation of *CDKI* genes in HCECs in vivo was made by immunohistochemistry in previous reports [23,24,26] and confirmed in the current study. It might be attributable to the limited HCECs in a single layer and limited number of donors. The residual part of corneal grafts after keratoplasty was collected over time, which assured the normality and number of endothelial tissues in some degree. Three to four corneas at similar ages were contained in each sample for RNA analysis in the present study, and a similar method was adapted in other experiments [41,42]. Although the characteristics of age-related decrease in ECD of rodents are observed to be similar [43,44], there are species-related differences in endothelial proliferative activity [23,45]. In addition, the major signaling pathway of cell senescence may differ among species, and the p16^INK4a^ mediated pathway appears to be more active in human beings [14]. All of the above indicates that the senescence mechanism of HCECs can not be deduced from the related studies in animals.

Senescence-related gene expression in HCECs ex vivo from the young and old donors was compared in this study. Immunohistochemistry and RT–PCR examinations confirm the expression of p16^INK4a^, p21^WAF1/CIP1^, p27^KIP1^, and p53 in HCECs. There was an increase in the mRNA expression level of *p16^INK4a^* in the old donors by quantitative real-time PCR whereas the levels of *p21^WAF1/CIP1^*, *p27^KIP1^*, and *p53* remained steady. An age-related increase in *p16^INK4a^* expression was also observed in other cell types in vivo [13] and HCECs cultured in vitro [26]. This reflects the stability of *p16^INK4a^* mRNA, which may be involved in the long-term cell cycle regulation. The p16^INK4a^ mediated signaling pathway might play an important role in HCEC aging. The age-related difference in *p16^INK4a^* expression may be used as an indicator for accelerated senescence of HCECs. As in other cell types [13], increased expression of *CDKI* genes was found with increasing age in the tubules of the kidney cortex. There were substantial differences between chronological and computed biological ages of the grafts, which showed an acceleration of senescence caused by the influence of various stresses in chronic allograft nephrology.

In the in vitro study, western-blot analysis showed that p16^INK4a^ and p21^WAF1/CIP1^ levels increased with donor ages in primary cultures of HCECs, but p27^KIP1^ levels did not have such an increase. However, p27^KIP1^ levels were significantly lower in the passage-4 HCECs from the older donors [26]. In the present study, the expression levels of *p21^WAF1/CIP1^* and *p27^KIP1^* genes did not differ with donor ages in the HCECs of ex vivo corneas. The results may reflect a difference in cellular response to stresses from the culture microenvironment rather than to an intrinsic age-related difference in *p21^WAF1/CIP1^* expression. Moreover, the *p27^KIP1^* expression level was considered to be different with multiple passaging for the difference between primary culture cells and passage 4 cells [26]. However, it is important to note that p27^KIP1^ protein expression is not solely dependent on its mRNA level but also regulated posttranslationally [46] although similar results can be observed ex vivo and in primary cultured HCECs. The mRNA level of the 30-year-old donors showed a significant increase among the eight age groups, which appeared to be an “outlier” (Figure 4). But for the same sample, the mRNA relative levels among the other groups were not consistent among the four genes. Such a difference might be attributed to the variation among samples. Similarly, there is at least a three-fold increase in *p27* mRNA in the 26-year-old group (Figure 5). Enlargement of the sample size might decrease the variation.

In this study, the *p53* expression level was not detected to be different between the young and old groups. Genes for some cell cycle control proteins such as *p53* are located close to the telomeres [47,48], and their expressions may be influenced by telomere length [19,20]. There was a correlation between the decreasing telomere length, the increasing level of p53, and the development of replicative senescence in bovine corneal endothelial cells in culture when the number of population doublings was increased to between 75 and 100. This may be the reason why neither telomere length nor p53 expression of HCECs from ex vivo corneas appeared to be different with age in the previous investigations and this study. In its role in the cell cycle control, p53 binds to and promotes the *p21^WAF1/CIP1^* gene. It is known that p53 is associated with a response to DNA damage either to inhibit cell division or to cause apoptosis. No age-related difference could be observed in the expression levels of p53 or p21^WAF1/CIP1^ in the HCECs from ex vivo corneas.

In the current study, we cannot rule out that organ procurement (harvesting and preservation) may have affected gene expression in the tissue samples although the result of the pathological examination was normal and the survival rate of endothelial cells was comparatively high. Tissue samples in vivo from healthy individuals would be preferable; however, since this may complicate the study design substantially, the current approach is widely accepted [49]. Moreover, further investigations with more cases are necessary to accurately define the exact type of relationship between expression and age. p16^INK4a^ expression can be used as an indicator for accelerated senescence of HCECs. The potential value of senescence-related genes in the development of late endothelial failure should be focused further. It is still unclear whether the different age-related responses of some *CDKI* genes are a result of environmental stresses or inherently determined by the age-related expression of p16^INK4a^. *CDKI* genes are involved in the negative regulation of cell cycle and suppression of HCEC proliferation, which may help to explore the methods to enhance the ECD of corneas ex vivo and in vivo.

In summary, p16^INK4a^, p21^WAF1/CIP1^, p27^KIP1^, and p53 are all expressed in HCECs despite donor ages. We conclude from the age-related increase in p16^INK4a^ expression that p16^INK4a^ signal pathway might play a key role in the process of senescence in HCECs.

## References

[1] Hoppenreijs VP, Pels E, Vrensen GF, Oosting J, Treffers WF (1992). Effects of human epidermal growth factor on endothelial wound healing of human corneas.. Invest Ophthalmol Vis Sci.

[2] Matsubara M, Tanishima T (1983). Wound-healing of corneal endothelium in monkey: an autoradiographic study.. Jpn J Ophthalmol.

[3] Laing RA, Sandstrom MM, Berrospi AR, Leibowitz HM (1976). Changes in the corneal endothelium as a function of age.. Exp Eye Res.

[4] Senoo T, Joyce NC (2000). Cell cycle kinetics in corneal endothelium from old and young donors.. Invest Ophthalmol Vis Sci.

[5] Zhu C, Joyce NC (2004). Proliferative response of corneal endothelial cells from young and older donors.. Invest Ophthalmol Vis Sci.

[6] Chen KH, Azar D, Joyce NC (2001). Transplantation of adult human corneal endothelium ex vivo: a morphologic study.. Cornea.

[7] Griffith M, Osborne R, Munger R, Xiong X, Doillon CJ, Laycock NL, Hakim M, Song Y (1999). Functional human corneal equivalents constructed from cell lines.. Science.

[8] Rieck PW, Stockhausen RM, Metzner S, Hartmann C, Courtois Y (2003). Fibroblast growth factor-2 protects endothelial cells from damage after corneal storage at 4 degrees C.. Graefes Arch Clin Exp Ophthalmol.

[9] Bourne WM, Hodge DO, Nelson LR (1994). Corneal endothelium five years after transplantation.. Am J Ophthalmol.

[10] Ing JJ, Ing HH, Nelson LR, Hodge DO, Bourne WM (1998). Ten-year postoperative results of penetrating keratoplasty.. Ophthalmology.

[11] Gong HQ, Gao H, Xie LX, Shi WY (2007). Ultrastructure changes in chronic corneal allograft dysfunction after penetrating keratoplasty.. Zhonghua Yan Ke Za Zhi.

[12] Patel SV, Hodge DO, Bourne WM (2005). Corneal endothelium and postoperative outcomes 15 years after penetrating keratoplasty.. Am J Ophthalmol.

[13] Chkhotua AB, Gabusi E, Altimari A (2003). Increased expression of p16(INK4a) and p27(Kip1) cyclin-dependent kinase inhibitor genes in aging human kidney and chronic allograft nephropathy.. Am J Kidney Dis.

[14] Halloran PF, Melk A, Barth C (1999). Rethinking chronic allograft nephropathy: the concept of accelerated senescence.. J Am Soc Nephrol.

[15] Joosten SA, Van Ham V, Nolan CE, Borrias MC, Jardine AG, Shiels PG, van Kooten C, Paul LC (2003). Telomere shortening and cellular senescence in a model of chronic renal allograft rejection.. Am J Pathol.

[16] Sherr CJ, Roberts JM (1999). CDK inhibitors: positive and negative regulators of G1-phase progression.. Genes Dev.

[17] Sharpless NE (2004). Ink4a/Arf links senescence and aging.. Exp Gerontol.

[18] Famulski KS, Halloran PF (2005). Molecular events in kidney ageing.. Curr Opin Nephrol Hypertens.

[19] Allsopp RC, Vaziri H, Patterson C, Goldstein S, Younglai EV, Futcher AB, Greider CW, Harley CB (1992). Telomere length predicts replicative capacity of human fibroblasts.. Proc Natl Acad Sci USA.

[20] Whikehart DR, Reigster SJ, Chang Q, Montgomery B (2000). Relationship of telomeres and p53 in aging bovine corneal endothelial cell cultures.. Invest Ophthalmol Vis Sci.

[21] Vaziri H, Benchimol S (1996). From telomere loss to p53 induction and activation of a DNA-damage pathway at senescence: the telomere loss/DNA damage model of cell aging.. Exp Gerontol.

[22] Brown JP, Wei W, Sedivy JM (1997). Bypass of senescence after disruption of p21^CIP1/WAF1^ gene in normal diploid human fibroblasts.. Science.

[23] Joyce NC, Navon SE, Roy S, Zieske JD (1996). Expression of cell cycle-associated proteins in human and rabbit corneal endothelium in situ.. Invest Ophthalmol Vis Sci.

[24] Joyce NC, Meklir B, Joyce SJ, Zieske JD (1996). Cell cycle protein expression and proliferative status in human corneal cells.. Invest Ophthalmol Vis Sci.

[25] Joyce NC, Harris DL, Mello C (2002). TGF-β and contact inhibition: Effects on expression of cclin-dependent kinase inhibitors in corneal endothelium.. Invest Ophthalmol Vis Sci.

[26] Enomoto K, Mimura T, Harris DL, Joyce NC (2006). Age differences in cyclin-dependent kinase inhibitor expression and Rb hyperphosphorylation in human corneal endothelial cells.. Invest Ophthalmol Vis Sci.

[27] Stocker FW, King EH, Lucas DO, Georgiade NA (1970). Clinical test for evaluating donor corneas.. Arch Ophthalmol.

[28] Xie LX, Kang F, Li G, Li Q, Yuan N (1986). Standards for the vitality of endothelial cells in donor grafts for penetrating keratoplasty.. Zhonghua Yan Ke Za Zhi.

[29] Mimura T, Joyce NC (2006). Replication competence and senescence in central and peripheral human corneal endothelium.. Invest Ophthalmol Vis Sci.

[30] Dong X, Xie L, Zhang X, Shi W, Li W, Yuan F (2000). A report on investigation and clinical application of corneal storage media.. Zhonghua Yan Ke Za Zhi.

[31] Mishima K, Handa JT, Aotaki-Keen A, Lutty GA, Morse LS, Hjelmeland LM (1999). Senescence-associated β-Galactosidase histochemistry for the primate eye.. Invest Ophthalmol Vis Sci.

[32] Bodnar AG, Ouellette M, Frolkis M, Holt SE, Chiu CP, Morin GB, Harley CB, Shay JW, Lichtsteiner S, Wright WE (1998). Extension of life-span by introduction of telomerase into normal human cells.. Science.

[33] Sigal SH, Rajvanshi P, Gorla GR, Sokhi RP, Saxena R, Gebhard DR, Reid LM, Gupta S (1999). Partial hepatectomy-induced polyploidy attenuates hepatocyte replication and activates cell aging events.. Am J Physiol.

[34] Ikebe H, Takamatsu T, Itoi M, Fujita S (1984). Cytofluorometric nuclear DNA determinations on human corneal endothelial cells.. Exp Eye Res.

[35] Ikebe H, Takamatsu T, Itoi M, Fujita S (1986). Age-dependent changes in nuclear DNA content and cell size of presumably normal human corneal endothelium.. Exp Eye Res.

[36] Ikebe H, Takamatsu T, Itoi M, Fujita S (1988). Changes in nuclear DNA content and cell size of injured human corneal endothelium.. Exp Eye Res.

[37] Bell KD, Campbell RJ, Bourne WM (2000). Pathology of late endothelial failure: late endothelial failure of penetrating keratoplasty: study with light and electron microscopy.. Cornea.

[38] Egan CA, Savre-Train I, Shay JW, Wilson SE, Bourne WM (1998). Analysis of telomere lengths in human corneal endothelial cells from donors of different ages.. Invest Ophthalmol Vis Sci.

[39] Ben-Porath I, Weinberg RA (2005). The signals and pathways activating cellular senescence.. Int J Biochem Cell Biol.

[40] Itahana K, Campisi J, Dimri G (2004). Mehanisms of cellular senescence in human and mouse cells.. Biogerontology.

[41] Paull AC, Whikehart DR. Expression of the p53 family of proteins in central and peripheral human corneal endothelial cells. Mol Vis 2005; 11:328–34. <http://www.molvis.org/molvis/v11/a38/> 15889017

[42] Ito K, Ito M, Elliott WM, Cosio B, Caramori G, Kon OM, Barczyk A, Hayashi S (2005). Decreased histone deacetylase activity in chronic obstructive pulmonary disease.. N Engl J Med.

[43] Fitch KL, Nadakavukaren MJ, Richardson A (1982). Age-related changes in the corneal endothelium of the rat.. Exp Gerontol.

[44] Meyer LA, Ubels JL, Edelhauser HF (1988). Corneal endothelial morphology in the rat. Effects of aging, diabetes, and topical aldose reductase inhibitor treatment.. Invest Ophthalmol Vis Sci.

[45] Joyce NC (2003). Proliferative capacity of the corneal endothelium.. Prog Retin Eye Res.

[46] Hengst L, Reed SI (1996). Translational control of p27Kip1 accumulation during the cell cycle.. Science.

[47] Levine AJ (1993). The p53 tumor suppressor gene and product.. Biol Chem Hoppe Seyler.

[48] Kaghad M, Bonnet H, Yang A, Creancier L, Biscan JC, Valent A, Minty A, Chalon P (1997). Monoallelically expressed gene related to p53 at 1p36, a region frequently deleted in neuroblastoma and other human cancers.. Cell.

[49] Melk A, Halloran P (2001). Cell senescence and its implications for nephrology.. J Am Soc Nephrol.

